# Supplementary material for: The genome sequence of the Pale Tussock moth, Calliteara pudibunda (Linnaeus, 1758) (Lepidoptera: Erebidae)
Source: Wellcome Open Res. 2025 Oct 21;10:587. [Version 1] doi: 10.12688/wellcomeopenres.25048.1 (PMC12963834; doi:10.12688/wellcomeopenres.25048.1)
Supplement: Peer review history

# Reviewer report: Reviewer response for version 1

**DOI:** 10.21956/wellcomeopenres.27614.r139896

**Published:** 2026-03-05

**Authors:** Annabel Whibley

**Affiliations:** 1 Bragato Research Institute, Clyde, New Zealand

Sivess and colleagues report the genome sequence of the Pale Tussock moth, *Calliteara pudibunda.* The sample collection, sequencing and assembly have been conducted under the Darwin Tree of Life project and follow standard processing pipelines and reporting templates. The Natural History section is particularly engaging.

 The assembly pipeline is appropriate, with good amounts of sequencing data obtained for each of the data tranches. The assembly is thoroughly evaluated, with a primary and alternative assembly presented. RNAseq data was recovered for the assembly backbone specimen but an annotation has not been released. The methods mention sample identity verification by barcoding but fail to specify whether both sequenced individuals were tested.

 As the only current representative of the genus, it is difficult to remark upon the atypical nature (or not) of the high chromosome count (n=88), and the HiC contact map looks very clean, but I would have liked to see some discussion of confidence of this karyotype, perhaps noting whether telomeric sequences have been recovered and/or how the recovered chromosomes correspond to Merian elements.  The Blob plot looks exceptionally clean (perhaps not surprising since >99.8% of the assembly was scaffolded to chromosomes) but some of those pseudo-chromosomes have “no database hit”, rather than “arthropod”, which is intriguing (based on n=80 arthropod, n=31 no hit).

Are sufficient details of methods and materials provided to allow replication by others?

Yes

Is the rationale for creating the dataset(s) clearly described?

Yes

Are the datasets clearly presented in a useable and accessible format?

Yes

Are the protocols appropriate and is the work technically sound?

Yes

Reviewer Expertise:

Genomics, Bioinformatics

I confirm that I have read this submission and believe that I have an appropriate level of expertise to confirm that it is of an acceptable scientific standard.

# Reviewer report: Reviewer response for version 1

**DOI:** 10.21956/wellcomeopenres.27614.r139897

**Published:** 2025-12-26

**Authors:** Henk den Bakker

**Affiliations:** 1 University of Georgia, Griffin, Griffin, USA

This data note describes the natural history and genome sequencing of the Pale Tussock moth, Calliteara pudibunda. The background/natural history section is well written as are the other usual Tree of Life sections.   

 I have two minor issues that should be addressed b ythe authors:

 1. (Background section) ...hop ( *Humulus lupulus*), a plant introduced to Britain by the early 16th century and extensively grown for beer production... Hop is native to Britain and was not introduced as is suggested here ( https://powo.science.kew.org/taxon/urn:lsid:ipni.org:names:303502-2), however, the cultivation of hops (and use in beer production) was introduced to Britain by the early 16th century. Please edit to reflect this.

 2. The data are based on two specimens: iCalPudi2 for genome sequencing and RNA-Seq, iCalPudi1 for Hi-C sequencing. If possible, please address why different specimens were used (e.g., not enough tissue) and if DNA barcoding for identification verification was performed on all specimens. I have reviewed multiple notes on moth genomes now and sometimes the same specimen is used for WGS and Hi-C sequencing, and sometimes multiple specimens are used.

Are sufficient details of methods and materials provided to allow replication by others?

Yes

Is the rationale for creating the dataset(s) clearly described?

Yes

Are the datasets clearly presented in a useable and accessible format?

Yes

Are the protocols appropriate and is the work technically sound?

Yes

Reviewer Expertise:

Bioinformatics, genomics, systematics

I confirm that I have read this submission and believe that I have an appropriate level of expertise to confirm that it is of an acceptable scientific standard.
